# Study on Pathogenesis of *Cytospora pyri* in Korla Fragrant Pear Trees (*Pyrus sinkiangensis*)

**DOI:** 10.3390/jof11040257

**Published:** 2025-03-27

**Authors:** Yiwen Zhang, Zhe Wang, Zhen Zhang, Lan Wang, Hongzu Feng

**Affiliations:** 1 College of Life Science and Technology, Tarim University, Alar 843300, China; 107572022301@stumail.taru.edu.cn (Y.Z.); wangzhe949510@163.com (Z.W.); 17591588959@163.com (Z.Z.); 2 Key Laboratory of Integrated Pest Management (IPM) of Xinjiang Production and Construction Corps in Southern Xinjiang, Tarim University, Alar 843300, China; 3 The National and Local Joint Engineering Laboratory of High Efficiency and Superior-Quality Cultivation and Fruit Deep Processing Technology of Characteristic Fruit Trees in Southern Xinjiang, Tarim University, Alar 843300, China

**Keywords:** pear *Valsa* canker, *Cytospora pyri*, infection process, pathogen identification

## Abstract

Pear *Valsa* cankers were found in various Korla fragrant pear orchards in Alaer, Xinjiang. Disease samples underwent tissue isolation, resulting in six isolates. Pathogenicity tests revealed that the XLFL-6 isolate was the most virulent, demonstrating typical *Valsa* canker symptoms. Research on its biological characteristics indicated that the optimal growth conditions for XLFL-6 were a temperature of 28 °C and a pH of five. Under these conditions, the colonies of XLFL-6 exhibited the largest growth diameter, and adding glucose and peptone separately to the Czapek medium was most conducive to the growth of its mycelium. Based on morphological observations and multigene sequence analyses (*ITS+TEF+TUB*), the pathogenic fungus was identified as *C. pyri*. The infection process of *C. pyri* was elucidated through tissue observations using both light and electron microscopy. The conidia displayed a similar germination pattern on both wounded and intact twigs. However, the infection process was delayed in the case of intact bark. By 8 h post-inoculation, the conidia achieved a germination rate of 15%. Although germination had occurred, the infection process had not yet commenced. In contrast, for wounded bark tissue, it was observed that 24 h post-inoculation, the fungal hyphae from the conidia directly invaded the wounded tissue. These hyphae penetrate the cell walls, proliferate within the host tissue, and spread throughout the phloem and xylem. After 20 d, numerous pycnidia had breached the bark surface, and yellow waxy gums filled with conidia flowed abundantly from the pycnidia ostioles, with the host tissue being nearly totally disintegrated. Regarding enzyme activity, the polygalacturonase (PG) activity, the primary cell wall-degrading enzyme in the treatment group, was seven times greater than that of the control group. The carboxymethyl cellulose (Cx) activity within the treatment group continued to increase. Xylanase activity rose swiftly to its peak between days 1 and 4, then decreased from days 5 to 10, although it remained higher than that of the control group. Overall, this study is the first to provide a detailed report on the characteristics and proliferation of *C. pyri* and further elucidates its modes and pathways of invasion.

## 1. Introduction

*Valsa* cankers are a destructive fungal disease of woody plants that can cause significant economic and ecological losses in orchards and forest ecosystems [1]. Among them, cankers caused by the fungus *Cytospora* spp. are prevalent and spreading rapidly in fragrant pear production areas [2]. China is one of the world’s three largest cultivated pear-growing centers [3]. Among its noteworthy varieties, the Korla fragrant pear holds significant economic importance in China and shapes the region’s fruit landscape [4,5]. However, in recent years, *Valsa* canker disease has emerged as a significant threat to the production of Korla fragrant pear. The disease mainly affects the lateral and main branches of the Korla fragrant pear tree, with the main symptoms being bark rot, necrosis, and tree weakness [6]. Severe cases of the disease can lead to the death of trees and the destruction of orchards, resulting in substantial economic losses and severely hampering the development of the Korla fragrant pear industry [7,8].

Although cankers are prevalent, *Cytospora* spp. are a weakly host-specific fungus, and there can be different pathogen species on the same host with the same symptoms [9,10,11,12]. The Korla fragrant pear tree canker fungus was identified as *Valsa ceratosperma* (Tode) Maire, the same as the apple tree canker fungus by Saito et al. [13]. Tang et al. [14] identified the Korla fragrant pear tree canker pathogen *Cytospora carphosperma* Fr. through morphological identification. Adams et al. [15] combined morphological and internal transcribed spacer region (*ITS*) sequence analysis methods to identify the Korla fragrant pear tree canker pathogen as *V. ceratosperma*. This shows that it is difficult to distinguish the pathogen species of Korla fragrant pear tree cankers through morphology alone. A single internal transcribed spacer region (*ITS*) sequence analysis of the pathogen has limitations, so it is unreliable to rely only on the host, morphological characteristics, or single-gene phylogenetic studies for the identification of Korla fragrant pear tree *Valsa* canker pathogens [16,17,18]. Thus, we cannot draw systematic and comprehensive conclusions on the Korla fragrant pear tree canker pathogen species in pear production areas. With the development of science and technology, the combination of the internal transcribed spacer region (*ITS*), transcription elongation factor-1α (*TEF*), and β-microtubulin (*TUB*) genes compensate for the one-sidedness of single genes, making the identification of balsam pear tree *Valsa* canker pathogens more reliable [19,20].

Regarding the infection mechanism of pathogenic fungi on host plants, it has been discovered that the pathogenic fungus *Valsa mali* displays only weak parasitism [21]. They mainly invade host plants through wounds caused by frostbite, sunburn, pruning, and other mechanical factors [22,23,24]. Scholars have also conducted relevant studies in this regard and found that the apple tree canker fungus *Valsa mali* var. *mali* in healthy branches could germinate but could not invade tissues. On the surface of wounded branches, the pathogen could be inside the tissues and cause tissue decay by Ke et al. [25]. Tamura et al. [26] found through a large number of section tests that during the dormant period, the pathogen expanded as individual mycelia in the bark tissues of apple trees, and the cells of host tissues in the site of mycelium expansion shriveled up and showed a slight brown discoloration. Annual isolated poplar branches were scalded and inoculated with *Cytospora chrysosperma* (Pers.) Fr. Two to three days after inoculation with the pathogen, a brown spot centered on the inoculation point formed on the surface of the branch and expanded; 7–9 d after inoculation, a ring-shaped spot was formed; 15–20 d after inoculation, a large number of pathogen propagules were produced on the surface of the branch [27].

On the other hand, in terms of the research on the pathogenic active substances of the *Valsa* canker pathogenic fungi, there are more studies concerning apple tree canker pathogens. These studies mainly focus on enzymes, degradation products of root bark glycosides, isocoumarins, and organic acids [28,29,30,31,32]. However, our understanding of cankers’ infestation process and pathogenic active substances in Korla fragrant pear trees is somewhat limited. In particular, the pathogen development within host tissues remains poorly understood. This lack of knowledge is not conducive to devising an effective control strategy for Korla fragrant pear tree canker disease. In this study, we aimed to address the issues of ambiguous pathogen identification, the unclear infestation process, and the mechanism of the pathogen regarding the Korla fragrant pear tree canker. Expressly, we set out to identify the Korla fragrant pear tree canker pathogen, ascertain its biological properties, and explore its infestation process and mechanism.

## 2. Materials and Methods

### 2.1. Sample Collection

In Xinjiang, China, six diseased samples were procured from pear trees afflicted with *Valsa* cankers within the Korla fragrant pear orchards situated in Nankou Town (geographical coordinates: 4°28′37″ N, 81°21′12″ E). Minuscule fragments of stem tissue (each measuring 0.5 × 0.5 cm) underwent surface sterilization via immersion in 1% sodium hypochlorite solution for 5 min, then they were rinsed three times with sterile water. Subsequently, the sterilized tissues were incubated in potato dextrose agar (PDA) medium under a controlled temperature of 28 °C for 5 d [33]. This process culminated in the acquisition of a total of 6 isolates exhibiting comparable morphological traits.

### 2.2. Pathogen Identification

Monospores of these isolates were purified and designated as XLFL-1 to XLFL-6 according to a previously described method. Subsequently, the purified isolate was inoculated onto PDA plates and incubated in a light-controlled chamber at 28 °C, with a photoperiod of 16 h of light and 8 h of darkness for 7 d. After this period, hyphal growth and colony color were examined. Additionally, morphological traits were examined, including growth rate, mycelial septation, conidial shape, and size [33,34].

The mycelia were obtained through the following procedures: firstly, a fungal layer was scraped from the periphery of a single test strain colony; secondly, it was transferred onto a PDA dish overlaid with sterile cellophane; and finally, it was incubated at 28 °C for 5 d [35,36]. Subsequently, the modified CTAB method was employed to extract genomic DNA from partial regions of four loci [37]. PCR was then utilized to amplify gene regions such as partial *ITS*, *TEF*, and *TUB* using the appropriate primer pairs (ITS1/ITS4 [38], EF1-728F/EF1-986R [39], and BT2a/BT2b [40]). For each gene, the PCR reaction mixture consisted of 1 μL of genomic DNA, 10.5 μL of ddH_2_O, 0.5 μL of each primer, and 12.5 μL of 2 × Taq Master Mix, yielding a final volume of 25 μL.

The procedure was as follows: initial denaturation at 94 °C for 5 min, followed by 30 cycles at 94 °C for 30 s, primer annealing at a suitable temperature for 30 s (58 °C for *ITS*, 52 °C for *TEF*, and 60 °C for *TUB*), primer extension at 72 °C for 45 s, and a final extension at 72 °C for 10 min. The amplification reaction is carried out on a Mastercycler pro-S gradient PCR instrument. The PCR product was visualized under UV light on a 1% agarose gel containing ethidium bromide (0.5 μg/mL), and amplicons were sequenced by Sangon Bioengineering Co. (Shanghai, China). The generated sequences were analyzed alongside related *Cytospora* taxa obtained from GenBank and recent publications. The alignment based on *ITS*, *TEF*, and *TUB* sequence data was performed using MAFFT v.6 and manually edited using BioEdit v.7.2.3. The sequences of *Diaporthe vaccinii* CBS 160.32 were included as an outgroup in all analyses. Phylogenetic analyses were performed with IQ-TREE v.1.6.12 for maximum likelihood (ML). Confidence levels for the nodes were determined using 1000 bootstrap replicates. Phylograms were plotted in FigTree v.1.4.3 and annotated in iTOL v6 (https://itol.embl.de/, accessed on 15 September 2024).

### 2.3. Pathogenicity Test

All six strains tested were identified as *C. pyri*. To prepare the conidia, *C. pyri* was cultivated on PDA under light at 28 °C until conidia formation. *C. pyri* inoculum was obtained by washing the spores with sterile water and adjusting the concentration to 1 × 10^6^ conidia per milliliter. In the inoculation process, one-year-old pear twigs measuring 25 cm in length were washed with tap water, immersed in 1% sodium hypochlorite for 1 min, and then rinsed 3 times with sterile water. Lastly, the ends of the twigs were sealed with wax. After charring each twig once using a flat iron (5 mm in diameter), the twigs were positioned horizontally in a plastic container. Subsequently, a suspension of 20 µL of conidia was carefully dripped onto the injured area of the branch and placed in an incubator at 28 °C for hydration [25,41].

### 2.4. Determination of Physiological Characterization

Among all the isolates, the most dominant and virulent isolate, XLFL-6, was selected for a detailed physiological study based on the pathogenicity test. To study the effect of temperature, a 10 μL spore suspension (1 × 10^6^ spores/mL) was inoculated on PDA plates and incubated at 18, 25, 28, 30, 36, and 40 °C. For the pH study, the PDA medium was adjusted to six different pH levels: 2, 3, 4, 5, 6, 7, and 8 by adding HCL or NaOH, and this was verified using a pH meter (Seven Compact TM S210, METTLER TOLEDO, Shanghai, China). To investigate the nutrient conditions, spore suspension was applied to PDA (potato dextrose agar), PMA (phorbol myristate acetate), SDA (Sabouraud dextrose agar), PSA (potato saccharose agar), and water agar medium, respectively. To elucidate the effect of the nitrogen source, Czapek medium was used as the base and incubated with equal amounts of sodium nitrate, ammonium sulfate, peptone, glutamic acid, asparagine, and a nitrogen-free supplement, respectively. For the carbon source study, spore suspension was incubated with equal amounts of starch, sucrose, glucose, maltose, lactose, and a carbon-free supplement, using Czapek as the basal medium. The diameter of fungal mycelium (mm) was measured at 12, 24, and 48 h, with three replicates established for each treatment.

### 2.5. Proliferation of Fungal Hyphae in Korla Fragrant Pear Trees

Conidia germination, pathogen infection, and pycnidia formation were examined using scanning electron microscopy (SEM). Bark sites inoculated with a conidia suspension were sampled at 4, 8, 12, 16, 20, and 24 h post-inoculation and 4, 10, 15, and 20 d post-inoculation. At each sampling point, both wounded and intact bark tissues were collected from the inoculation sites. For SEM analyses, the specimens were cut into 3 × 3 mm pieces and treated as detailed by Kang and Buchenauer [42]. Specimens were fixed in 4% glutaraldehyde diluted with 0.1 mol/L phosphate buffer (pH = 6.8) overnight at 4 °C, rinsed with the same buffer for 1 min, dehydrated in a graded series of ethanol concentrations (30, 50, 70, 80, 90, and 100%) for 30 min each, subjected to critical point drying, mounted on stubs, and sputter-coated with a gold/palladium target. The specimens were observed with Thermo Fisher Apreo SEM (Thermo Fisher Scientific, Waltham, MA, USA) at 15 KV, and the experiment was repeated 3 times.

To study *C. pyri* colonization in pear bark tissues via light and transmission electron microscopy (TEM), bark sections with visible canker lesions were sampled after 4, 10, 15, and 20 d. Each sample was collected from five different lesions and processed for light microscopy. For paraffin sections, we followed Naifu’s method with modifications [43]. Specimens were fixed in FAA for 48 h, dehydrated using an ethanol gradient, and clarified in ethanol and xylene. The treated material was soaked in wax for coating and then cut into 10 µm transverse and longitudinal slices using a microslicing machine. After the sections were dewaxed using a xylene and ethanol gradient, they were placed in a toluidine blue solution and stained for 15 min. The stained slides were sealed with Canada balsam, dried, and photographed using an optical microscope (Olympus BX43, Olympus Corporation, Tokyo Metropolis, Japan).

TEM was conducted following the method of Kang and Buchenauer [42]. Samples were initially treated with a solution of 4% glutaraldehyde in a 0.1 mol/L phosphate buffer (pH = 6.8) overnight and then washed with the same buffer. Subsequently, they were exposed to a 1% osmium tetroxide solution, dehydrated using a series of ethanol concentrations, and infiltrated with a mixture of LR white resin and ethanol in various ratios followed by pure resin. Each infiltration step lasted for a minimum of 6 h, with the final resin infiltration lasting 72 h. The specimens were then encased in capsules and polymerized at a temperature of 55 °C over 2 d. Following contrast enhancement with uranyl acetate and lead citrate, the samples were examined using JEOL 1230 TEM (JEOL Japan Electronics Co., Ltd., Musashino, Japan) operating at 80 KV.

### 2.6. Cell Wall Degradation Enzyme Activity Assay

Ten μL of spore suspension (1 × 10^6^ spores/mL) was inoculated into the branches and humidified at 25 °C. Branches exhibiting spots were removed and drained of water, and 4 mL of the extract was added per gram. Subsequently, an appropriate amount of quartz sand was added, grinding at low temperatures, and centrifuged at 8500 rpm for 15 min at 4 °C. The supernatant was utilized to determine enzyme activity. The activity of cell wall degradation enzymes was determined using ultraviolet–visible spectrophotometry. For the determination of the activities of PG, Cx, and xylanase, the extinction value of the reaction mixture was measured using dinitrosalicylic acid (DNS) at 540 nm, and the enzyme activity was calculated based on the amount of reducing sugar released by the enzymatic reaction. The substrate used for the Cx activity assay was 1% carboxymethyl cellulose (CMC), the substrate for PG was also 1% CMC, and the substrate for xylanase was 0.5% xylan. The unit of enzyme activity is defined as 1 μmol of reducing sugars (U/mL or U/mg total proteins) from the catalytic substrate per milliliter of enzyme solution (per milligram of enzyme protein) per minute at 50 °C.

## 3. Results

### 3.1. Isolation and Morphological Characterization

**Figure 1:**
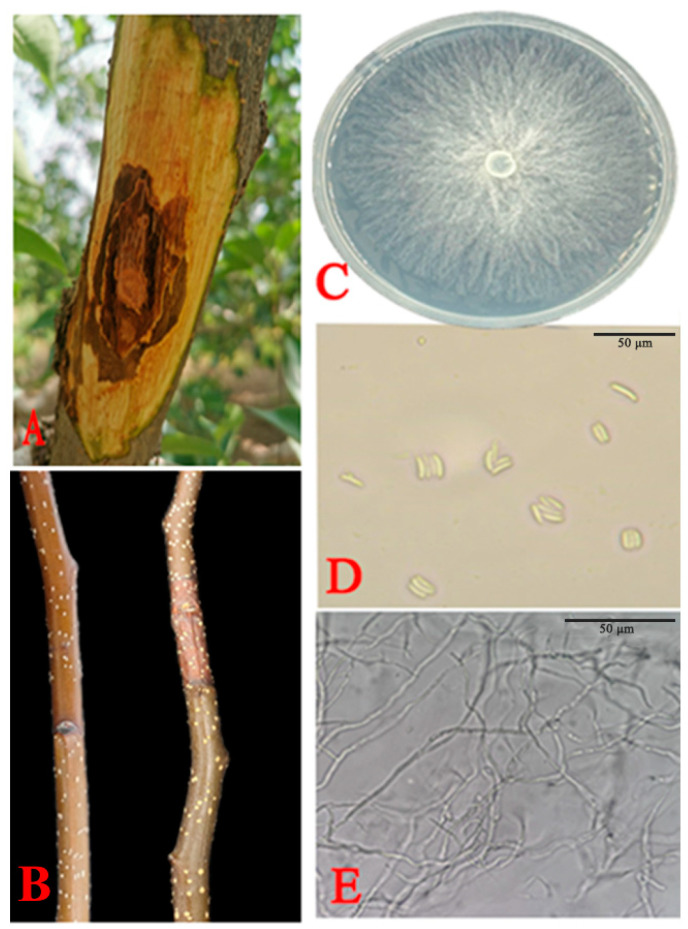
Morphological features of *C. pyri* isolated from Korla fragrant pear branches. (**A**) Field symptoms of an infected branch with pear *Valsa* canker. (**B**) Inoculated Korla fragrant pear branches exhibiting rot symptoms at 28 °C. (**C**) Morphological characteristics of colonies on PDA after 7 d. (**D**) Conidia. (**E**) Fungal hyphae. Scale bars: (**D**,**E**) = 50 µm.

A total of six *Cytospora* fungal isolates were obtained from the infected Korla fragrant pear branches (Figure 1A). The isolates grew well (8 mm per day at 28 °C) on the PDA medium and showed the same morphological appearance. All the isolates formed white mycelia after 7 d at 28 °C. All the representative isolates of the fungal hyphae are elongated, multitudinously branched, and have a septum. Their conidia were hyaline, allantoid, and 3.55–6.35 × 0.55–1.45 µm in size (Figure 1C–E).

### 3.2. Pathogenicity

After 7 d of incubation, brown lesions appeared on the branches inoculated with the six representative strains. Control shoots inoculated with water agar showed no lesions. Isolate XLFL-6 was the most common lesion around the inoculation point, and XLFL-6 was the most virulent pathogen. Based on the morphological and molecular characterization, the reisolated pathogen from the Korla fragrant pear sprigs resembled the original isolate (Figure 1B).

### 3.3. Molecular Sequencing and Phylogenetic Analysis

DNA analysis was utilized to categorize the samples further. The corresponding sequences were identical following sequencing and alignment, indicating that the six selected samples belonged to identical species. The rDNA-*ITS*, *TEF*, and *TUB* gene sequences of XLFL-1 and XLFL-6 were submitted to the NCBI, and the following accession numbers were obtained: XLFL-1 (Accession No. PP494037, PP505998, and PP506000) and XLFL-6 (Accession No. PP494040, PP505999, and PP506001).

**Figure 2:**
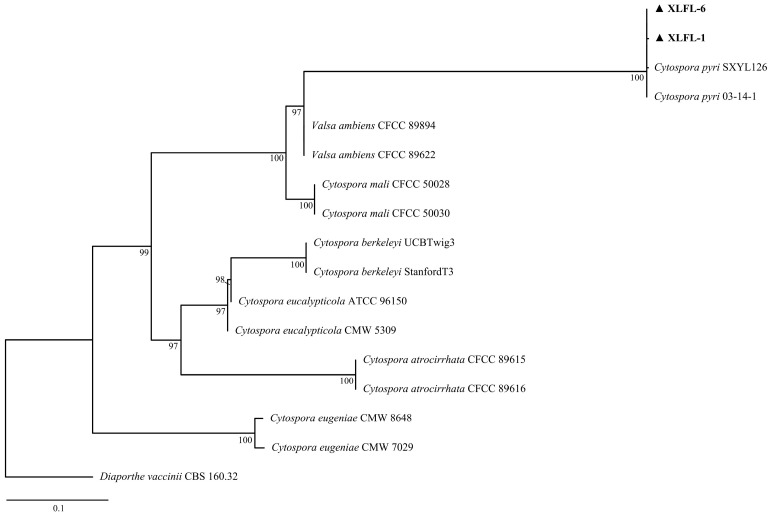
Phylogenetic analyses of the combined sequence alignment of *ITS*, *TEF*, and *TUB* isolates and reference strains were performed with IQ-TREE v.1.6.12 for maximum likelihood (ML). XLFL-1 and XLFL-6 are the two most representative isolates of all isolates numbered.

The two isolates’ rDNA-*ITS*, *TEF*, and *TUB* gene sequences were analyzed via BLAST (https://blast.ncbi.nlm.nih.gov/Blast.cgi, accessed on 21 August 2024) and IQ-TREE v.1.6.12 software for homology and phylogenetic analyses. The BLAST searches against the NCBI database revealed that the *ITS* sequence had a 99.83% homology (PP494037 and PP494040), the *TEF* gene had a 99.22% homology (PP505998 and PP505999), and the *TUB* sequence had 99.57 and 100% homologies (PP506000 and PP506001) with *C. pyri* sequences. A phylogenetic analysis showed a 100% bootstrap support value, indicating that XLFL-1 and XLFL-6 were *C. pyri* (Figure 2).

### 3.4. Physiological Characterization

**Figure 3:**
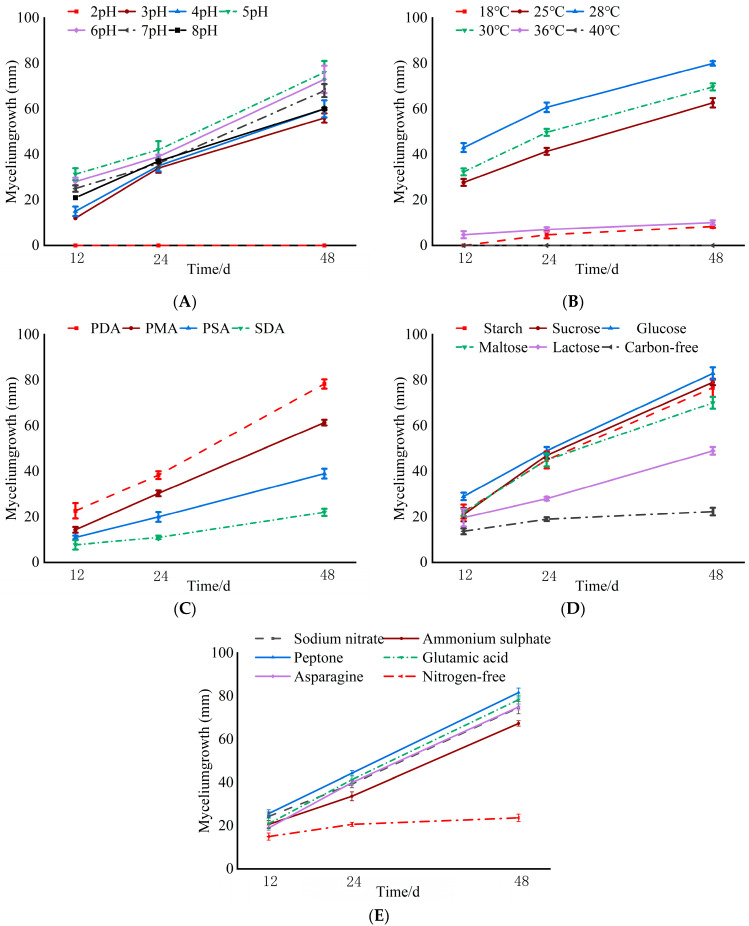
Effects of different culture conditions on mycelium growth of *C. pyri*. (**A**) pH. (**B**) Temperature. (**C**) Culture medium. (**D**) Carbon source. (**E**) Nitrogen source.

The data presented in Figure 3A reveal the differences in the colony growth of *C. pyri* at various pH levels. When the pH value was five, the fungal mycelium exhibited the maximum radial growth, reaching 76 mm, followed by that at a pH of six (73 mm). When the pH was two, the fungal mycelium was unable to grow.

Isolate XLFL-6 was capable of growing at different temperatures varying from 18 °C to 36 °C. It grew well at 28 °C, achieving a growth diameter of 87 mm, followed by that at 25 °C (84 mm). At 40 °C, XLFL-6 failed to grow, and this temperature seemed to be the thermal inactivation point of this isolate. Temperatures ranging from 25 °C to 28 °C were optimal for the growth and sporulation of *C. pyri* (Figure 3B).

Isolate XLFL-6 was able to grow in different media, namely PDA, PMA, PSA, and SDA. At 48 h, XLFL-6 grew better in the PDA medium, reaching a growth diameter of 78 mm. It did not grow as well in the PMA and PSA media, with growth diameters of 61 mm and 39 mm, respectively, and grew more slowly in the SDA medium (Figure 3C).

The fungal mycelium was able to utilize different carbon sources (namely starch, sucrose, glucose, maltose, lactose, and carbon-free sources) and different nitrogen sources (including sodium nitrate, ammonium sulfate, peptone, glutamic acid, asparagine, and nitrogen-free sources). The growth conditions were better when glucose was used as the carbon source, with a growth diameter of 83 mm, followed by sucrose and starch as the carbon sources. The growth was worse under the lactose and carbon-free conditions. Among the nitrogen sources, the mycelium grew well when peptone was used as the nitrogen source, reaching a growth diameter of 81 mm, followed by glutamic acid and sodium nitrate, with growth diameters of 78 mm and 74 mm, respectively. The mycelium grew slowly under the nitrogen-free condition (Figure 3D,E).

### 3.5. Conidia Germination and Invasion Mode

**Figure 4:**
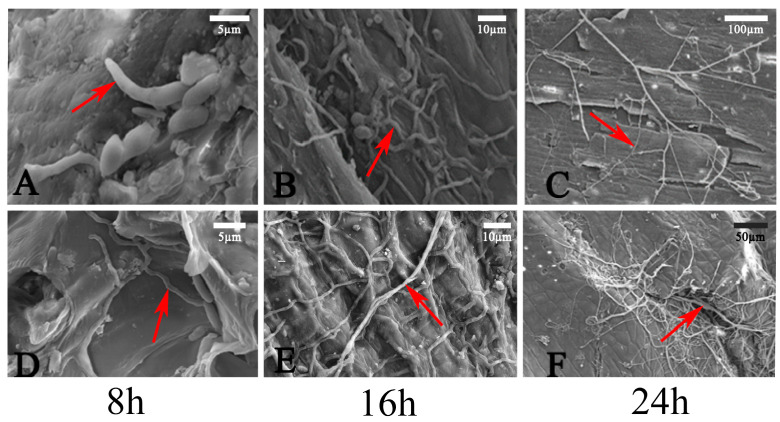
SEM analysis. (**A**–**C**) Fungal hyphae on the surface of healthy bark at 8 h, 16 h, and 24 h. (**D**–**F**) Fungal hyphae germinating on the surface of wounded bark at 8 h, 16 h, and 24 h. Red arrows indicate fungal hyphae. Scale bars: (**A**,**D**) = 5 µm; (**B**,**E**) = 10 µm; (**C**) = 100 µm; (**F**) = 50 µm.

After the conidia suspension was inoculated onto the surfaces of burned and intact branches, the conidia germination and invasion mode were studied via SEM. On the uninjured shoots’ surface, the conidia’s germination rate on the bark surface was 15% at 8 h. After 16 h, the conidia shriveled, and the fungal hyphae became sparser. At 24 h, a small number of hyphae adhered to the surface of the shoots, with the ends of the fungal hyphae gradually tapering off, and no invasion of the fungal hyphae into the host tissue was observed (Figure 4A–C). On the surface of the injured shoots, the germination rate of conidia was 75% after 8 h, and the average length of the bud tube was 20 μm. At 16 h, coarse hyphae appeared on the shoots’ surface, and many fungal hyphae were observed entering the host from the wound at 24 h. These results indicated that the germination rate of *C. pyri* on the wounded shoots was higher than that on the non-injured shoot surface, and the growth rate of hyphae on the bark surface of the wounded shoots was higher than that on the surface of the uninjured shoots. Moreover, fungal hyphae could only enter the interior of the shoot through the wound and did not possess the ability to directly penetrate the surface of the shoot and enter its internal tissues (Figure 4D–F).

### 3.6. Expansion Process Within Bark Tissue

**Figure 5:**
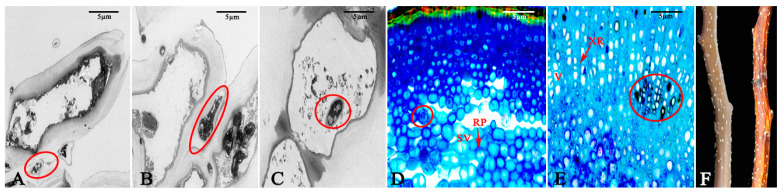
Expansion of hyphae of the pathogen in host tissues. (**A**) The fungal hyphae parasitize between the host cells after 24 h. (**B**) The fungal hyphae penetrate the host cells after 48 h. (**C**) The fungal hyphae enter the interior of the host cells after 96 h. (**D**) The fungal hyphae spread in the phloem after 4 d. (**E**) The fungal hyphae spread in the xylem after 10 d. (**F**) The branches in a healthy state and those after being diseased for 10 d. The red circle indicates the fungal hyphae. RP: phloem ray parenchyma; SV: sieve tube; V: xylem vessels; XR: xylem ray cells. Scale bars: (**A**–**F**) = 5 µm.

The expansion of fungal hyphae within the bark was examined using TEM, which revealed the aggressive colonization of host cells by these hyphae. After 24 h, the fungal hyphae primarily spread intercellularly among the cortical parenchyma cells as the fungus expanded into the intercellular spaces. This expansion induced alterations in the host cells, leading to the separation of the plasma membrane. The phenomenon of plasma membrane separation following pathogen infection indicates that the host cell exhibits a response upon contact with the pathogenic mycelium, signifying an active stress response to the infection. However, this antagonistic reaction did not inhibit the invasion of the pathogenic mycelium; after 48 h, the mycelium could penetrate the host cell wall and grow, resulting in some deformation of the host cells. By 96 h, the hyphae of the pathogen had successfully entered the host cells and proliferated, leading to the degradation of the host cell wall and significant deformation of the host cells (Figure 5A–C). Paraffin sections revealed that the hyphae of the pathogen only expanded within the phloem by 4 d, but their penetration depth could reach the xylem after 10 d (Figure 5D,E). Compared to healthy shoots, the surface of the branches after 10 d exhibited severe shrinkage, and reddish-brown lesions appeared on all of the branches (Figure 5F).

### 3.7. Pycnidia Development Process

**Figure 6:**
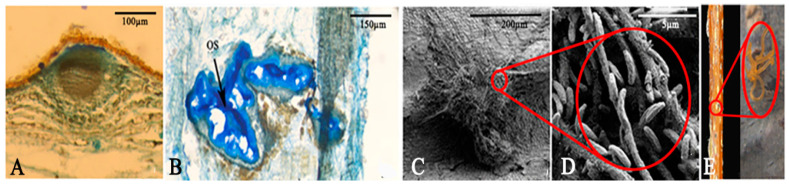
Structural features of pycnidia. (**A**) Immature pycnidia after 15 d. (**B**) Mature pycnidia after 20 d. (**C**) Fungal hyphae from the pycnidia. (**D**) Conidia attached to the fungal hyphae. (**E**) Cirrus produced on the surface of the branch after 20 d. The black arrow indicates the ostiole. OS: ostiole. Scale bars: (**A**) = 100 µm; (**B**) = 150 µm; (**C**) = 200 µm; (**D**) = 5 µm.

Fifteen d after inoculation, as observed by paraffin sections, a dense intertwining of the fungal hyphae was noted at the center of the fungal hyphae cluster, leading to dark brown spots on the twigs’ surfaces. Paraffin sections indicated that pycnidia had initially formed; they were spherical and embedded in the phloem tissue. After 20 d, when the pycnidium had matured, longitudinal sections revealed that the pycnidium comprised many locules with one ostiole (Figure 6A,B). The pycnidia had emerged from the bark surface, and a viscous gel containing conidia was exuded through the pycnidia ostiole, resulting in a substantial accumulation of conidia on the fungal hyphae (Figure 6C,D). Under a light microscope, it could be seen that the cirrus broke through the bark and grew, displaying an orange-yellow color and irregular entanglement (Figure 6E).

### 3.8. Changes in Cell-Wall-Degrading Enzyme Activity During C. pyri Infection of Korla Fragrant Pear Branches

**Figure 7:**
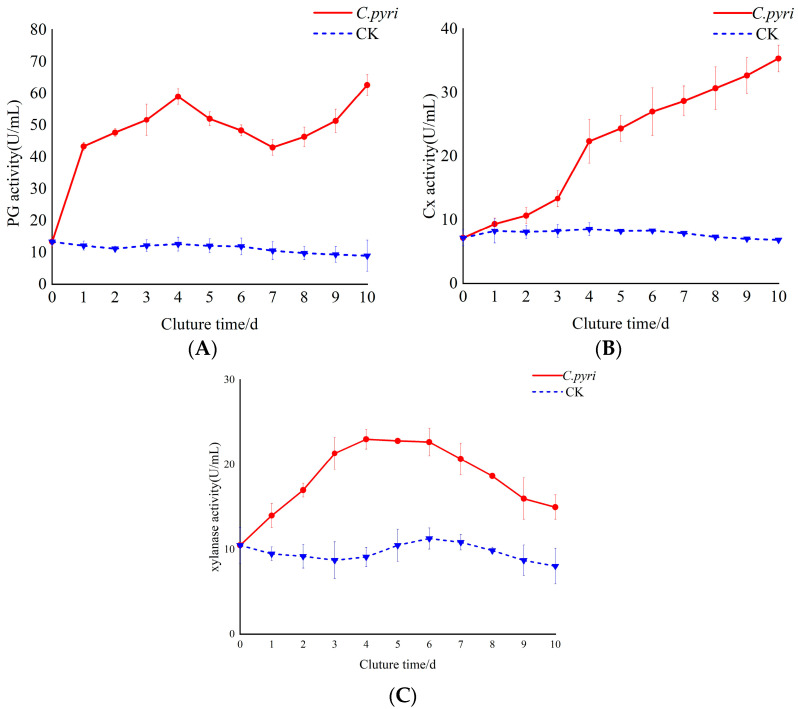
Changes in the activity of cell-wall-degrading enzymes in the branches of Korla fragrant pear trees after inoculation with *C. pyri*. (**A**) Determination of PG enzyme activity in the branch tissue infected by *C. pyri*. (**B**) Determination of Cx enzyme activity in the branch tissue infected by *C. pyri*. (**C**) Determination of xylanase enzyme activity in the branch tissue infected by *C. pyri*. *C. pyri*: Treatment group, treated with *C. pyri*. CK: Control group, treated with sterile water.

It can be observed from Figure 7A that the PG activity of *C. pyri* was significantly higher than that of the control within 1–10 d after *C. pyri* infection. The overall trend was characterized by an initial increase, a decrease, and another increase. Specifically, the PG content exhibited an upward trend during the first 1–4 d, a downward trend after 4 d, and an upward trend again after 7 d. Moreover, the activity reached its maximum value at 10 d, which was seven times that of the control group. As depicted in Figure 7B, in the early stage of infection (1–2 d), the activity of Cx in the treatment group showed little difference from that of the control group. However, at 4 d, the Cx activity of the treatment group was significantly higher than that of the control group. Furthermore, within 1–10 d, the Cx activity in the treatment group continued to increase, while the Cx activity of the control group remained relatively stable. As shown in Figure 7C, the activity of xylanase increased rapidly during 1–4 d and reached a peak at 4 d, which was 2.5 times higher than that of the control group. From 5 to 10 d, the xylanase activity demonstrated a downward trend yet remained higher than that of the control group.

## 4. Discussion

Recently, pear *Valsa* cankers have been increasingly impacting large-scale Korla fragrant pear cultivation. This disease has a rapid onset and high transmissibility, thus becoming one of the major diseases afflicting pears. Moreover, accurately identifying pathogenic fungi remains challenging [12,44]. We isolated and purified a pathogen from Korla fragrant pear trees and conducted a preliminary study on the pathogen *C. pyri* in Korla pear tree disease in China from multiple aspects, including its morphology, multigene sequence, phylogeny, physiology, and proliferation in host tissues. *C. pyri* was a reported pathogen causing pear disease, and the culture characteristics and morphological characteristics of this strain are similar to those of *Cytospora* spp.

Based on the physiological studies, potato dextrose agar (PDA) medium is the most suitable medium for its growth. A temperature of 28 °C is optimal for *C. pyri*. In comparison, it fails to grow at 40 °C, indicating that overly high temperatures are not conducive to its growth and reflecting the close correlation between its physiological metabolic process and temperature [45]. *Cytospora* species can grow under a pH ranging from two to eight, which shows that *Cytospora* species cankers can adapt to ecological environments with different pH levels to a certain extent. The optimal pH range is five to six, suggesting that an acidic environment is more favorable for the growth of pathogenic mycelia. This might be because the optimal pH for most *Cytospora* species is mostly acidic, and the amplification and expression of acidic exoproteases, such as glutamic acid protease G01 family-related genes, may be the determinants influencing the adaptation of decay pathogens to the host or environment and their ability to infect bark [46]. There were differences in the utilization capacities of carbon sources and nitrogen sources. Among the carbon sources, glucose boasted the highest utilization rate, while among the nitrogen sources, peptone had the highest utilization rate. The fact that glucose can rapidly supply energy to fungi implies that *C. pyri* has developed a high adaptability to high-efficiency energy substances. The efficient utilization of peptone suggests a sophisticated amino acid transport and metabolism system within its cells. This indicates that the fungus has a high degree of adaptability to its environment and can decompose organic substances more rapidly, thus having a competitive advantage.

To clarify the germination process of *C. pyri* conidia on their host as well as the process of invasion into host tissues, we employed SEM. It was observed that conidia could usually germinate on the epidermis of both intact and wounded pear branches, yet the germination rates differed significantly. On the epidermis of wounded pear branches, the germinated conidia could invade the host tissues directly through the wounds. In contrast, no invasion by the germ tube was witnessed on the intact bark surfaces, indicating that infection depends on the presence of wounds. Green mold is generally a severe post-harvest disease of citrus fruits. *P. digitatum* can invade the fruit tissues only when bruised or wounded [47]. The hyphae could not penetrate the epidermal cell wall of the host tissue. This is probably because the multiple exoenzymes secreted by *C. pyri* could not soften the protective epidermis of Korla fragrant pear bark but could readily decompose the necrotized cells. When the host suffers mechanical damage (such as that caused by pruning, insect damage, or drought/freezing stress), the pathogen can invade through these wounds and spread into the host tissues, ultimately resulting in canker lesions. These findings imply that the control strategy for *Valsa* cankers should focus on enhancing tree vigor and reducing injury and pruning wounds to prevent the tree from being infected in the first place.

It was observed that the fungal hyphae of *C. pyri* initially grew in the intercellular space and then extended to the cell wall and the interior of the host cell. The growth within the host would, to some extent, lead to the deformation of cells and the enlargement of organelles, ultimately resulting in the disintegration and death of the host cells, giving rise to branch cankers. Our observations of tissue samples with canker lesions showed that the fungal hyphae could infiltrate host tissues beyond the canker boundary as early as 4 d after inoculation. Moreover, within 10 d, they could break down the cambium and penetrate the xylem vessels. This rapid proliferation is similar to the behavior demonstrated by other pathogens that cause cankers, such as *Cytospora cincta*, which is responsible for peach cankers [48]. Likewise, apple *Valsa* cankers also infected trees through natural bark crevices and wounds caused by adverse climatic conditions, pruning, and other mechanical injuries. The infected hyphae grew intercellularly and intracellularly and extensively penetrated the phloem and xylem of trunks and main forks. Consequently, pear *Valsa* cankers were difficult to control with fungicides. This was due to the fact that they could penetrate the xylem tissue and that the pear *Valsa* canker pathogens had latent infection characteristics [49,50]. In previous studies, it has been found that cell wall degradation enzymes are directly involved in the pathogenic process. Among them, pectin decomposition enzymes are one of the significant factors contributing to *Valsa mali*-induced plant cankers. Highly active pectinase was detected in the branch tissues infected by *C. pyri*, which can dissolve the colloidal layer in the cell wall and destroy the cell protoplasm [51]. Recent research findings have demonstrated that *Pythium myriotylum*, a necrotrophic oomycete, acts as the causal agent causing soft rot in various crops [52]. The efficient colonization ability of necrotrophs relies heavily on the secretion of diverse plant cell-wall-degrading enzymes (CWDEs), which play a crucial role in the pathogenic process [53]. Moreover, researchers have also confirmed through the study of genes related to the pathogenic enzymes of pathogenic bacteria that pectinase is one of the core virulence factors of pathogens causing plant diseases. For instance, Chen et al. [54] reported that RsPG2, a polygalacturonic acid gene in rice blast, could regulate the effect of polygalacturonase by modulating polygalacturonase degradation in rice tissues to induce necrotic symptoms. Cho et al. [55] knocked out the pectate lyase gene PL1332 from *Alternaria brassicicola*, significantly reducing its virulence.

This study discovered that the PG content was the highest among the three enzymes, namely PG, Cx, and xylanase. Additionally, the PG activity continued to increase when the hyphae expanded from the intercellular space to the inside of the host cell during the first 4 d. The main function of PG is to hydrolyze the cell wall, which leads to the disintegration of the cell structure, resulting in tissue softening and decay [56]. This implies that PG might play a role in the pathogenesis of *Valsa* canker pathogens. The Cx activity was relatively low in the early stage of infection. However, when the hyphae entered the host cell, the Cx activity increased significantly. When the pathogen first enters the host, its growth and reproduction activities are focused on survival and the initial colonization, and the plant cell wall cellulose is not exposed in large quantities. Once the hyphae break through the cell wall, the cellulose substrate becomes abundant, and nutrients are sufficient, thus promoting a significant increase in cellulase activity [57]. During the first 4 d, the activity of xylanase continued to increase. This could be because when *C. pyri* infects Korla pear branches, xylan is one of the main components of the plant cell wall. Microorganisms destroy the cell wall structure, facilitating the invasion of plant cells by pathogenic fungi and thereby promoting the colonization and expansion of pathogenic fungi in plants. For example, *Fusarium graminearum* secretes xylanase to degrade the cell wall when it infects wheat ears [58]. From 5 to 10 d, the synthesis of xylanase might be inhibited due to changes in the intracellular environment, such as nutrient consumption and metabolite accumulation, and the degradation rate of enzymes is relatively accelerated, resulting in a gradual decrease in enzyme content and activity.

## 5. Conclusions

The pathogen identified as the cause of *Valsa* cankers in Korla fragrant pear trees has been determined to be *C. pyri* through a morphological analysis and combined multigene phylogenetic analysis. The growth of *C. pyri* was optimally enhanced by the addition of glucose and peptone to PDA medium, maintained at 28 °C and with a pH that was adjusted to five. The mechanisms of infestation by *C. pyri* were explored using a combination of paraffin sections, scanning electron microscopy, and transmission electron microscopy. It was found that *C. pyri* could not directly penetrate the bark to invade the inner tissues of healthy one-year-old isolated branches of the Korla fragrant pear tree. Instead, *C. pyri* invaded the branches through wounds, subsequently spreading and proliferating within the bark tissues, which led to the development of canker symptoms and the production of pycnidia after 20 d. Polygalacturonase (PG) plays a significant role in the infestation of the branches of Korla fragrant pear trees by *C. pyri*.

## Data Availability

The original contributions presented in this study are included in the article. Further inquiries can be directed to the corresponding authors.
